# Development and pilot testing of a National Men’s Health Index

**DOI:** 10.1186/1472-6963-14-S2-P126

**Published:** 2014-07-07

**Authors:** Chin Hai Teo, Chirk Jenn Ng

**Affiliations:** 1 Department of Primary Care Medicine, University of Malaya, Kuala Lumpur, Malaysia

## 

Recent men’s health reports from Asia, Australia, Canada and Europe have consistently shown that men have higher morbidity and mortality compared to women in most health conditions. There are numerous factors that may contribute to this and they range from men’s behaviour, socio-economic to health system factor. To date, there is no systematic way to document and compare men’s health determining factors and their impacts on men’s health. Policy makers do not have proper guidelines to accurately identify and prioritize on the key factors that affect men’s health status in individual country.

To overcome this problem, we propose the concept of the National Men’s Health Index (NMHI), which aims to assess men’s health status and its social health determinants of a country. Men’s health status consists of several categories including survivability, physical and mental health, which is divided further into indicators such as life expectancy, communicable, non-communicable diseases, injuries and suicide rate. The overall NMHI score indicates the wellbeing of men in the country while the sub-score will provide an indication of physical and mental wellbeing. The social health determinants are factors that influence the NMHI score and they are made up of lifestyle risk factors, socio-economy status, safety, environmental and health system, which are measured by parameters such as literacy rate, smoking prevalence, pollution index and health expenditure.

The NMHI will be developed systematically in 4 steps. Firstly, two systematic reviews will be carried out to review the existing composite health and non-health indices as well as to identify established indicators of men’s health. Secondly, NMHI model will be developed based on the systematic reviews and the expert opinions. Thirdly, a Delphi survey will be conducted with men’s health key opinion leaders in the world to prioritize the men’s health indicators. Fourthly, the NMHI model will be revised and weighted accordingly before pilot testing.

The NMHI scores of each country will be ranked and this will be correlated with the various social health determinants to explain the score. We believe that NMHI can serve as a guide for policy makers to identify gaps in men’s health and help them to prioritize health policy for men in their country. The NMHI will also allow countries to share experiences and effective strategies with one another and to monitor men’s health progress.

